# Antinociceptive and anxiolytic-like effects of *Lavandula angustifolia* essential oil on rat models of orofacial pain ^*^

**DOI:** 10.1590/1678-7757-2002-0304

**Published:** 2023-01-06

**Authors:** Vanessa Bordenowsky Pereira LEJEUNE, Raphael Vieira LOPES, Darciane Favero BAGGIO, Laura de Oliveira KOREN, Janaina Menezes ZANOVELI, Juliana Geremias CHICHORRO

**Affiliations:** 1 Universidade Federal do Paraná Departamento de Farmacologia Curitiba Paraná Brasil Universidade Federal do Paraná (UFPR), Setor de Ciências Biológicas, Departamento de Farmacologia, Curitiba, Paraná, Brasil.

**Keywords:** Nasal administration, Postoperative pain, Nociceptive orofacial pain, Anxiety, Hyperalgesia

## Abstract

Nociceptive and inflammatory orofacial pain is highly prevalent in the population, which justifies the search for safer analgesics. There is increasing evidence of the analgesic and anxiolytic potential of *Lavandula angustifolia* essential oil (LAV EO), which may represent, when administered through inhalation, may represent a safer alternative for pain treatment.

**Objective:**

to evaluate whether LAV EO has antinociceptive effect in the formalin test, and anti-hyperalgesic and anxiolytic-like effects in rats subjected to a model of orofacial postoperative pain.

**Methodology:**

Female Wistar rats were exposed to LAV EO (5%) by inhalation for 30 minutes. After exposure, animals were injected with formalin (2.5%, 50 μL) or saline into the hind paw or upper lip and the number of flinches or facial grooming time, respectively, were evaluated. Likewise, on day 3 after intraoral mucosa incision, the animals were exposed to LAV EO and facial mechanical, and heat hyperalgesia were assessed. The influence of LAV EO inhalation on anxiety-like behavior was assessed in operated rats by testing them on the open field (OF) and elevated plus maze (EPM).

**Results:**

LAV EO reduced the phase II of the paw formalin test and both phases of the orofacial formalin test. On day three post-incision, LAV EO reduced heat and mechanical hyperalgesia, from 30 minutes up to three hours, and reduced the anxiety-like behavior in operated rats without causing locomotor deficit.

**Conclusion:**

LAV EO inhalation results in antinociceptive and anxiolytic-like effects in orofacial pain models, which encourages further studies on LAV EO indications and effectiveness on orofacial pain conditions.

## Introduction

The orofacial region encompasses many unique structures, including the cornea, meninges, teeth, nasal and oral mucosa and temporomandibular joint, which are frequently afflicted by painful disorders.^1^ Acute trigeminal pain is highly frequent even after minor oral surgical procedures, and it markedly affects patient recovery and quality of life.^2^ In fact, acute trigeminal pain has been associated to emotional co-morbidities, especially anxiety, which has been considered a contributing factor for pain chronification.^3^ Moreover, the prevalence of orofacial pain has been estimated twice as high in women compared with men, and female sex has also been suggested a risk factor for orofacial pain chronification.^3 , 4^

The formalin test, applied to the orofacial region or hind paw of rats, has been widely used for the screening of compounds with analgesic potential. Regardless of the injection site, formalin induces a biphasic response, consisting of a brief acute phase, due to direct activation of peripheral nociceptors, followed by an inflammatory pain phase.^5^ Inflammatory pain may also be assessed in rodents by incision surgery models, which mimic post-operative pain. In the intraoral mucosa incision model, our previous studies demonstrated that 3 days after surgery rats show spontaneous pain, heat and mechanical hyperalgesia, and anxiety-like behavior.^6^ Thus, these models allow the pre-clinical investigation of antinociceptive, anti-hyperalgesic and anxiolytic effect of test substances.

*Lavandula angustifolia* has been used whole or as an essential oil (EO) for centuries for a variety of therapeutic purposes.^7 - 11^ The anxiolytic effect of *Lavandula angustifolia* essential oil *(* LAV EO) has been extensively demonstrated^8 , 12 - 15^ In fact, inhalation of LAV EO has been shown to reduce peri-operative anxiety in patients undergoing oral surgery.^16 - 19^ On the other hand, the analgesic effect of LAV EO is less studied, and most studies that addressed this topic used the oral route of administration.^20^ For instance, oral administration of LAV EO reduced both phases of the paw formalin test in rats. However, the inhalation of certain EO allows a safe, simple, non-invasive and low-cost therapeutic alternative that may be useful in some clinical contexts. This route may be of special interest in orofacial pain conditions since the EO may have a rapid direct effect on peripheral trigeminal afferents, as well as a rapid transportation from the nasal mucosa to the brain through the olfactory and trigeminal nerves.^21^

In light of these considerations, this study sought to investigate the antinociceptive and anxiolytic effects of LAV EO administered by inhalation in rats subjected to orofacial pain. Firstly, we assessed the effect of LAV EO by inhalation in the paw and orofacial formalin test. The first served as a control and allowed the comparison between LAVOE effect in a trigeminal versus a non-trigeminal innervated region. Then, we explored the effect of LAV EO in facial heat and mechanical hyperalgesia and anxiety-like behavior associated with facial post-operative pain.

## Methodology

### Animals

Experiments were conducted on 250 adult female Wistar rats (Rattus norvegicus) weighting 180 to 220 g. The number of animals per group was determined through a pilot study and the G*Power software. Rats were housed four per cage in a climate-controlled room at 22±2°C on a 12-hour light/dark cycle (lights on at 7:00 am) with food and water *ad libitum* and wood shavings changed on alternate days. The procedures were conducted during the day, between 8 AM and 6 PM, and the animals were acclimated for at least 48 hours before each experiment. The animals were provided by the bioterium of the Federal University of Parana and all protocols were previously approved by the Research Ethics Committee for the Use of Animals in the Biological Sciences Sector of the Federal University of Paraná (CEUA/BIO-UFPR #1348), all in accordance with the Brazilian guideline of the National Council for the Control of Animal Experimentation (CONCEA) and the Animal Research: Reporting of In Vivo Experiments (ARRIVE) guidelines.

### Drugs and solutions

LAV EO and neutral vegetable oil (i.e. avocado oil) were obtained from a commercial source (Quinarí, Ponta Grossa, Brazil) and both were diluted in cereal alcohol (Cloroquímica, Curitiba, Brazil) at a concentration of 5% (v/v) following previous studies.^8^ The same batch of LAV EO was used in all experiments, which was evaluated by gas chromatography-mass spectrometry (GC/MS, provided by Quinarí) and presented 30% of linalool and 40% of linalyl acetate as main constituents, which is in accordance with the pattern stablished by ISO-3515:2002.^22^ Ketamine hydrochloride (50 mg/kg; Syntec, São Paulo, Brazil) and Xylasin hydrochloride (7 mg/kg; Syntec, São Paulo, Brazil) were used as anesthetics. Some experiments were carried out with formalin administration (Alphatec, Brazil) diluted to 2,5% in 0,9% sterile saline solution (Equiplex Pharmaceutical Industry, Goiania, Brazil). Naltrexone (from Tocris Bioscience, Bristol, UK) was diluted in saline sterile solution and the dose used was based on previous studies.^23^ All drugs were freshly prepared just before the experiments.

### Exposure to LAV EO

**Figure 1:**
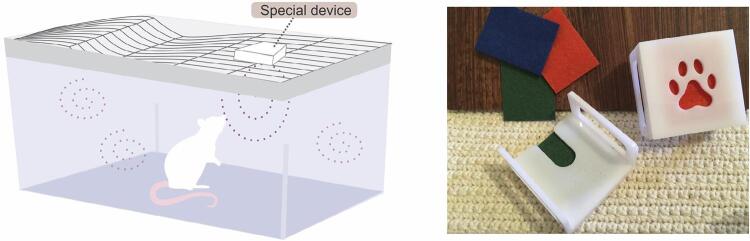
Special device made of acrylic for LAV EO or vehicle inhalation (5%). The felt received 0,5 ml of LAV EO or vehicle (avocado oil) and it was replaced for each animal. The front opening allows standardized volatilization of the LAV EO

Exposure to LAV EO or vehicle (avocado oil 5% — VEH) was done individually in a cage box (41x32x16,5 cm), with a special device (as shown in Figure 1 ) for standardized volatilization, allowing the animals to inhale the EO for 30 minutes, following previous studies.^8 , 12^ The animals were kept in separate rooms during the experiments so that the control group was not exposed to LAV EO odor. Thus, the interference of the essential oil on the control group was avoided.

### Formalin Test

The orofacial formalin test was conducted as previously described by our group.^24^ The formalin test was also conducted via injection in the plantar surface of one hind paw as described previously by Abbott, Franklin and Westbrook^25^ (1995). Rats were placed individually in cages for an acclimatization period in order to minimize any stress-related behavioral changes for about 15 minutes. The animals received a subcutaneous (s.c.) injection of formalin (2.5%, 50 μL) or vehicle (50 μL of saline) into the upper lip or hind paw. The facial grooming (i.e. time spent rubbing the injected area with its forepaws) time was evaluated for 30 minutes, and the flinches number (i.e. shaking of the injured paw) was counted for 60 minutes, respectively. The first and second phases of the orofacial formalin test were considered 0 to 3 minutes and 12 to 30 minutes after injection, respectively, whereas after formalin injection in the hind paw, the first phase was considered 0 to 5 minutes, and the second phase 15 to 60 minutes.

### Postoperative pain model

The postoperative pain was induced based on the method proposed by Urata, et al. and further characterized by our group.^6 , 26^ Rats were anesthetized with a mixture of ketamine and xylazine (50 mg/kg and 7 mg/kg, i.p., respectively). The rats were kept with the mouth open and placed in a lateral position. An incision was made in the intraoral mucosa, always on the right side (depth, 2 mm; length, 10 mm). One suture was placed in the middle of the incision and the rats were accompanied until their recover from anesthesia. The sham group was subjected to the same manipulation, but incision and suture were not performed. On day 3 after the procedures (i.e. sham or incision), facial heat and mechanical hyperalgesia and anxiety-like behavior was assessed, as described below.

### Assessment of heat hyperalgesia

Facial heat hyperalgesia was evaluated in rats as previously reported by group.^6 , 26^ Firstly, rats were habituated to the restraining method to avoid stress during the test. Facial heat sensory threshold was assessed by the approximation of a radiant heat source (about 50°C) 1 cm from the surface of the right vibrissa whisker pad area. The response latency to display either head withdrawal or vigorous flicking of the snout was recorded. A cut-off time of 20 seconds was established to prevent tissue damage.

### Assessment of mechanical hyperalgesia

Rats were maintained individually for habituation into observation cages for at least 2 hours before the test. The mechanical threshold was measured using a graded series of 8 von Frey filaments ranging from 0,04 to 8,0 g in increasing order (Semmes-Weinstein monofilaments, Stoelting, Wood Dale, IL — USA) as described previously by our group.^6^ The test consisted of three consecutive applications of each Von Frey filament into the rat’s vibrissae pad on the same side of intraoral mucosal incision or sham procedure, with an interval of 30 seconds between each application. Each stimulation series began with the filament producing the lowest force and proceeded up to the filament that evoked two positive responses, including brisk head withdrawal, facial grooming, or sharp escape or attack reactions against the filament. To avoid unspecific responses, a baseline measure was performed and only animals that did not respond to the application of the 8 g filament were included in the study.

### Open Field Test (OF)

This test was conducted as previously described by our group^27^ and consisted in placing the rats at the center of an arena (50 cm long, 50 cm wide, and 40 cm high) with closed sidewalls and nine quadrants floor. Rats’ behavior was recorded for 5 minutes for posterior evaluation of the number of intersections between these units (crossed squares) and the number of crossings in the central quadrant with the four paws. The first measurement was taken as an index of locomotor activity and the second one as index of anxiety-like response.

### Elevated Plus Maze Test (EPM)

This test was performed following previous studies^28^ and was carried out in a light-controlled room (60 lux). The test began by placing the animal in the center of the apparatus facing the open arm and, for 5 minutes, the number of entries and time spent on the open and closed arms was recorded. In addition, ethological measures, including risk assessment and head dip, were evaluated.^29^ As anxiety index, we evaluated open arm time and entries (in percentage): percentage of open arm entries (%OAE=100×open arm entries/total entries) and percentage of time spent on the open arms (%OAT=100 × time spent on open arms / [time spent on open arms + time spent on closed arms]). In addition, the time of risk assessment(s) and frequency of head dip was also used as anxiety index. The anxiolytic-like effect is considered when the animal makes more entries and spends more time in the open arms, causing the time of the risk assessment to decrease and the frequency of head dip to increase. As index of locomotor activity, the frequency of entry in the enclosed arms was quantified.

### Experimental protocols

Paw and orofacial formalin test: The animals were exposed to VEH or LAV EO 5% for 30 minutes and, immediately after the inhalation, received formalin or vehicle injection in the plantar surface of one hind paw, and the flinches were counted for 60 minutes. An independent group of rats was subjected to the same procedures, but after inhalation they received an injection (s.c.) of formalin or saline into the upper lip and facial grooming time was evaluated for 30 minutes.

Postoperative orofacial pain model: Evaluation of mechanical and heat hyperalgesia was carried out in independent groups of rats. The baseline responses to heat and mechanical stimulus were assessed and rats were subjected to intraoral mucosa incision or sham procedure. On day 3 after the surgery, the animals were exposed to LAV EO or VEH for 30 minutes, followed by assessment of facial heat and mechanical hyperalgesia at 30 minutes after inhalation and at 1 hour-interval until the 4th hour.

EPM and OF tests: On day 3 after intraoral mucosa incision or sham procedure, the animals were exposed to LAV EO or VEH for 30 minutes, followed by the EPM test, and briefly transferred to the OF apparatus. The tests were used in sequence as already describe by group.^30^ The same animals were recorded for 5 minutes in each apparatus for posterior analysis of their behavior.

### Statistical Analysis

The Kolmogorov-Smirnov normality test was applied to ensure that the data met the criteria for performing parametric tests. When the criteria were accepted, the results were expressed as mean ± standard error of the mean (SEM) of 10 animals per group. Two-way analysis of variance (ANOVA) with repeated measures (RM) was used to analyze data from the formalin test (hind paw and orofacial region) and mechanical and heat hyperalgesia induced by intraoral incision. Cumulative data of phases I and II of the formalin test and data from the OF and EPM tests were analyzed by one-way ANOVA. When appropriate, the ANOVAs were followed by Bonferroni post hoc test and the level of significance was set at P<0.05. Statistical analysis was performed using GraphPad Prism 6 for Windows (GraphPad Software, San Diego California USA).

## Results

### LAV EO reduces formalin-induced nociceptive behavior

**Figure 2:**
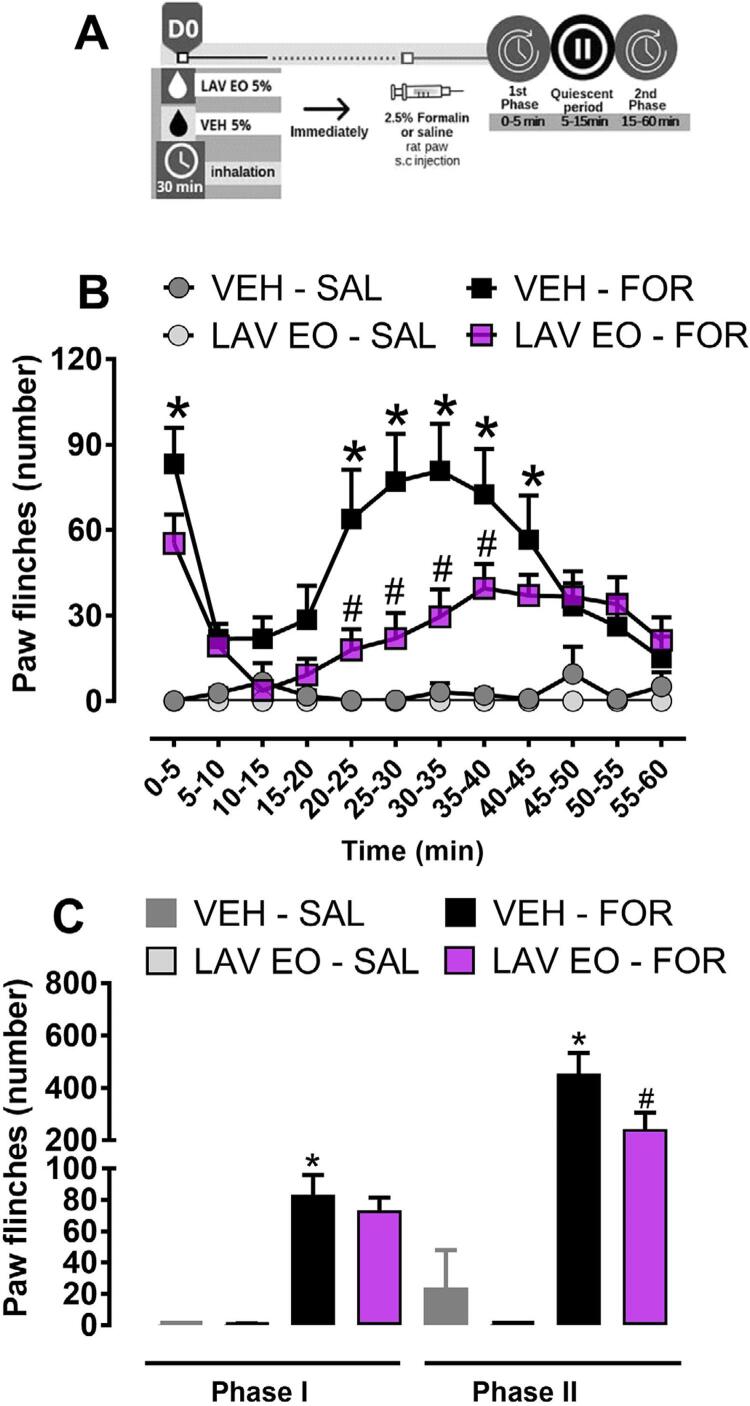
LAV EO reduces formalin-induced nociceptive behavior in the rats’ hind paw. (A) Timeline of the experimental procedures. (B) Rats were exposed to LAV EO or VEH, both at 5% concentration, for 30 minutes followed by Formalin (FOR, 2.5%/50 μL) or Saline (SAL, 50 μL) injection into the rats’ hind paw. The number of flinches was recorded for 60 minutes. (C) Effect of LAV EO in phase I (0–5 minutes) and Phase II (15–60 minutes) of formalin-induced nociceptive responses. Data are expressed as mean ± SEM (n=10). *P<0.05 when compared with VEH-SAL group and #P<0.05 when compared with VEH-FOR group

Two-way ANOVA with RM showed statistical difference in the treatment (F (3, 36)=22.80, P<0,05) and time (F (11, 396)=7.375, P<0.05) factors. Also, we observed an interaction of these factors (F (33, 396)=4.889, P<0.05). As can be seen in the Figure 2B , post hoc test showed that Formalin (2.5%, 50 μL) injected in the rats’ hind paw induced a biphasic nociceptive response, which was significantly different from saline-injected rats (P<0.05). The treatment (LAV OE) did not reduce the number of flinches at 0–5 min (phase I, P>0.05). However, in the 20–40 minutes interval, the previous exposure to LAV EO reduced the number of flinches, indicating antinociceptive effect (P<0.05). Figure 2C illustrates the cumulative nociceptive response in phase I (0–5 minutes) and phase II (15–60 minutes) of the formalin test (hind paw). One-way ANOVA demonstrated differences between groups in the phase I (F (3, 36)=35,46, P<0.05) and phase II (F (3, 36)=16,38, P<0.05). Post hoc test showed that previous exposure to LAV EO induced antinociceptive effect only in the phase II (P<0.05).

**Figure 3:**
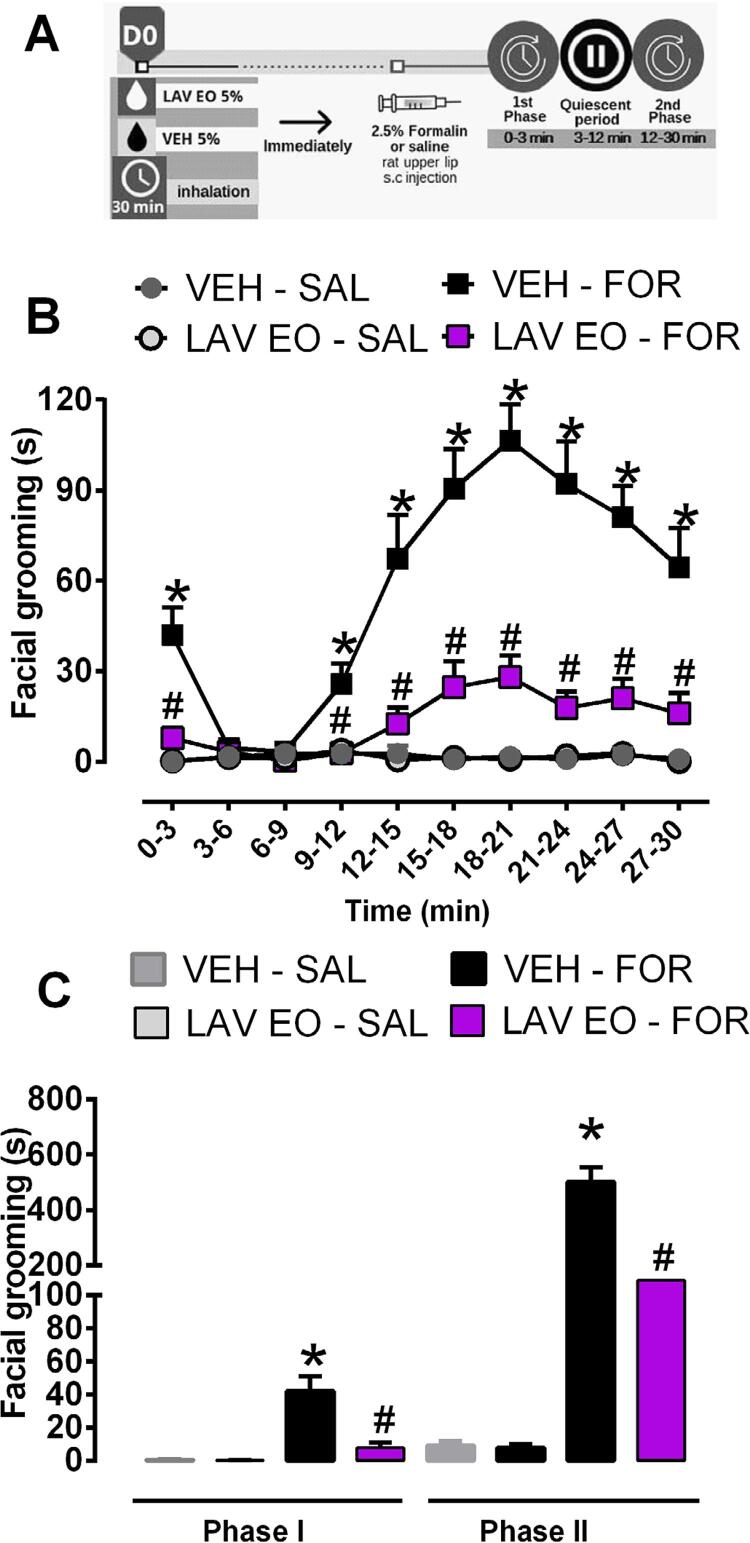
LAV EO reduces formalin–induced nociceptive behavior in the orofacial region. (A) Timeline of the experimental procedures. (B) Rats were exposed to LAV EO or VEH, both at 5% concentration, for 30 minutes followed by Formalin (FOR, 2.5%/50 μL) or Saline (SAL, 50 μL) injection into the upper lip. The grooming response was recorded for 30 minutes. (C) Effect of LAV EO in phase I (0–3 minutes) and Phase II (12–30 minutes) of formalin-induced facial grooming. Data are expressed as mean ± SEM (n=10/group). *P<0.05 when compared with VEH-SAL group and #P<0.05 when compared with VEH-FOR group

As shown in Figure 3B , two-way ANOVA with RM showed differences (treatment factor: F (3, 36)=50.53, P<0.05; time factor: F (9, 324)=21.47, P<0.05). Post hoc analysis showed that subcutaneous injection of formalin (2.5%, 50 μL) into the upper lip induced a biphasic nociceptive response, which was significantly different from saline-injected rats (P<0.05). Also, the treatment (LAV OE) at 0–3 min and 9–30 minutes of interval caused a significant reduction in the facial grooming time, indicating antinociceptive effect (P<0.05). As can be seen in the Figure 3C , one-way ANOVA showed differences between experimental groups (Phase I: F (3, 36)=16.39, P<0.0001 and Phase II F (3, 36)=54.23, P<0.05). Post hoc test evidenced significant antinociceptive effect in both phases after inhalation of LAV OE (P<0.05), differently of previous exposure to LAV EO in saline-injected rats that did not show difference in the phase I or II (P>0.05).

### LAV EO reduces heat and mechanical hyperalgesia in the postoperative orofacial pain model in rat

**Figure 4:**
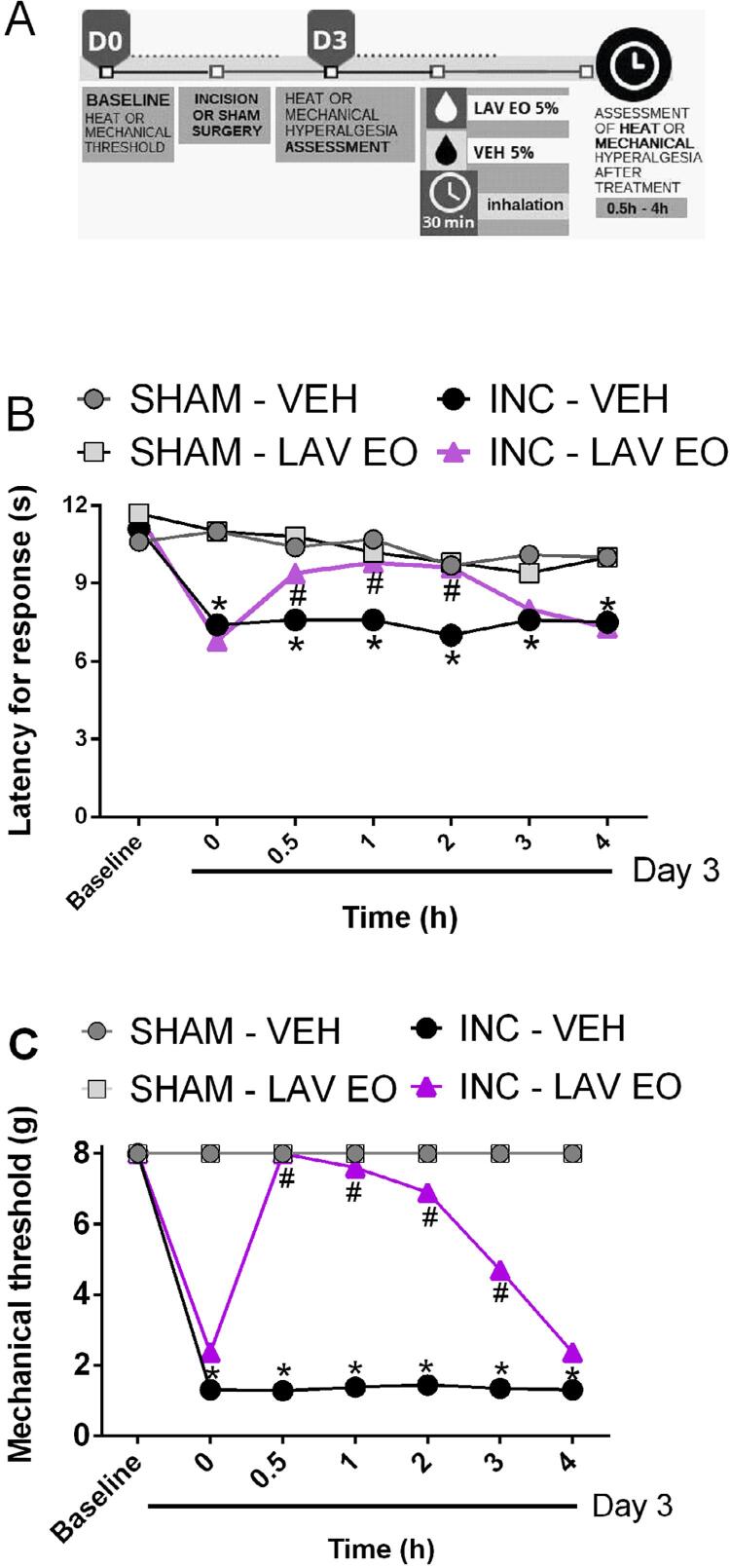
LAV EO reverses heat and mechanical hyperalgesia in the postoperative orofacial pain model in rats. (A) Timeline of the experimental procedures. On day 3 after the oral incision rats developed heat and mechanical hyperalgesia which were significantly reduced by animals’ exposure to LAV EO (5% for 30 minutes) compared to the vehicle-exposed group (B and C, respectively). Data are expressed as mean ± SEM (n=10). *P≤0,05 compared with SHAM-VEH group; and #P≤0,05 compared with INC-VEH group

As shown in Figure 4B , two-way ANOVA with RM showed statistical significance on treatment (F (3, 16)=20.28, P<0.05) and time (F (6, 96)=36.14, P<0.05) factors besides interaction between these two factors (F (18.96)=9.670, P<0.05). Post hoc analysis showed that rats subjected to LAV EO by inhalation presented increased latency for response to the heat stimulus starting in 30 minutes and persisting up to 2 hours after treatment (P<0.05), characterizing an antihyperalgesic effect. Previous exposure to LAV EO in the sham group did not show difference in the latency of response when compared to sham rats exposed to vehicle ( *i.e.* , SHAM-VEH, P>0.05). In addition, two-way ANOVA with RM also demonstrated a significant effect when facial mechanical hyperalgesia induced by intraoral incision was evaluated ( Figure 4C - treatment factor: F (3, 36)=210.8, P<0.05; time factor: F (6, 216)=88.48, P<0.05; interaction factor: F (18, 216)=54.45, P<0.05). Post hoc test showed that LAV EO attenuated facial mechanical hyperalgesia induced by intraoral incision, with a significant effect that lasted from 30 minutes up to 3 hours (P<0.05). Before the incision or sham surgery, results showed no significant differences between the groups in the baseline (P>0.05) and previous exposure to LAV EO in sham rats did not alter the mechanical threshold compared to the control group ( *i.e.,* SHAM-VEH, P>0.05).

### LAV EO presented an anxiolytic effect in rats subjected to intraoral incision

**Figure 5:**
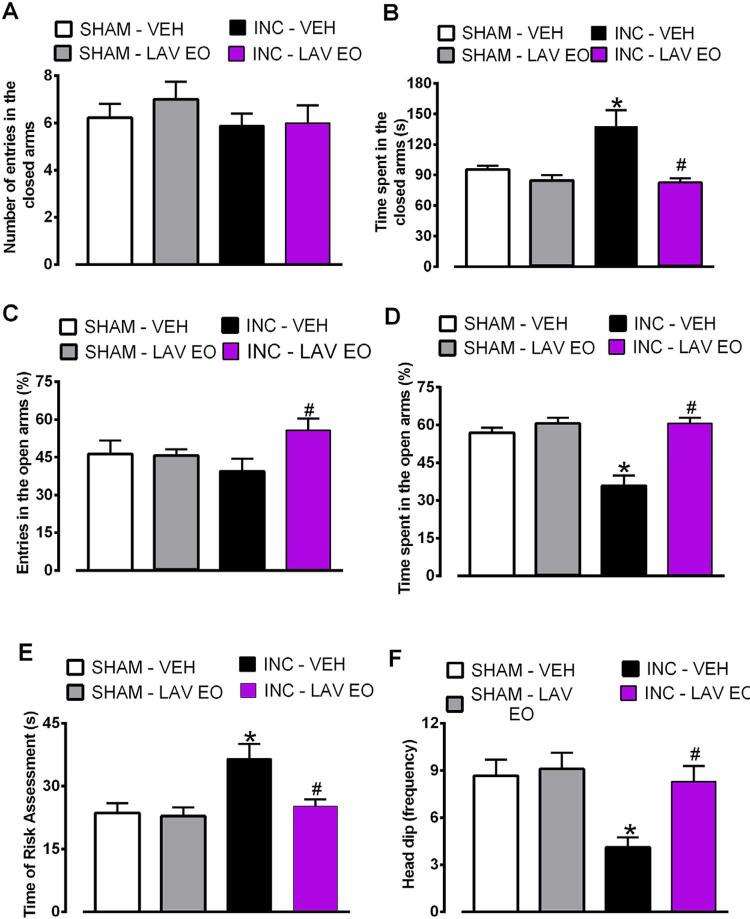
LAV EO causes anxiolytic effect in rats subjected to the intraoral incision and evaluated in the Elevated Plus Maze. (A) Effect of LAV EO in the number of entries in the closed arms; (B) Effect of LAV EO in time spent in the closed arms; (C) Effect of LAV EO in the number of entries in the open arms; (D) Effect of LAV EO on the time spent in the open arms; (E) Effect of LAV EO in stretch (risk assessment) movements; (F) Effect of LAV EO in the number of head dip. Data are expressed as mean ± SEM (n=9–10). *P≤0,05 compared with SHAM-VEH group; and #P≤0,05 compared with INC-VEH group

As shown in Figure 5 , when we analyzed parameters from closed arms (panels A and B) one-way ANOVA did not reveal difference between the groups on number of entries (F (3, 34)=0,5784, P>0.05), indicating absence of locomotor deficit; but revealed an effect when time spent in these arms was analyzed (F (3, 34)=9,081, P<0.05). Post hoc test showed that incision rats presented an increase in the time spent in these arms, indicative of anxiety-like effect. Interestingly, the LAV EO exposure in rats with incision was able to block this anxiety-like effect (P<0.05). In the other measures taken from the EPM ( Figure 5 - panels C, D, E, and F) one-way ANOVA revealed a statistically significant effect [entries in the open arms (%): F (3, 34)=5,051, P<0.05; time spent in the open arms (%): F (3, 34)=18,33, P<0.05; time of risk assessment: F (3, 34)=6,502, P<0.05; frequency of head dip: F (3, 34)=5,754, P<0.05]. Post hoc analysis showed that for entries and time spent in the open arms, rats with incision, compared to sham animals, presented a decrease in these parameters (P<0.05), indicating an anxiety-like effect. However, this effect was reversed in incision animals exposed to LAV EO (P<0.05). In addition, this anxiety-like effect of incision was also demonstrated on risk assessment and head dip measures, compared to sham animals (P<0.05). We highlight that these ethological analyses demonstrate the capability of the LAV EO to reverse this anxiety-like effect in the incision animals by decreasing risk assessment (P<0.05) and increasing the head dip frequency (P<0.05).

**Figure 6:**
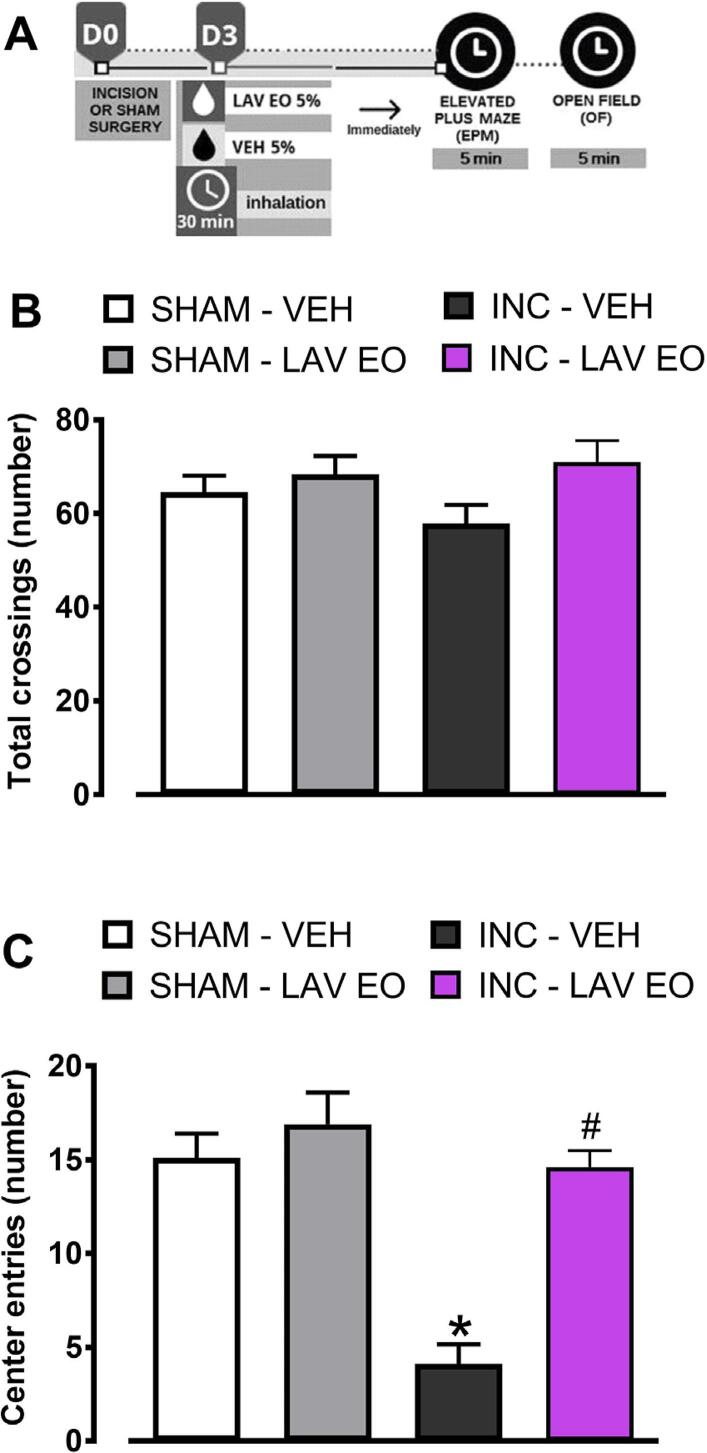
LAV EO causes anxiolytic effect in rats subjected to the intraoral incision and evaluated in the Open Field test. (A) Timeline of the experimental procedures; (B) Effect of LAV EO in the number of crossings; (C) Effect of LAV EO in the number of entries in the center of the arena. Data are expressed as mean ± SEM (n=9–10). One–way ANOVA followed by Bonferroni's post hoc test. *P≤0,05 compared with SHAM-VEH group; and #P≤0,05 compared with INC-VEH group

One-way ANOVA confirmed that the number of crossings in the open field did not differ between the groups (F (3, 34)=1,981, P>0.05; Figure 6B ), indicating absence of locomotor deficit. However, one-way ANOVA indicated difference when number of entries in the center of the arena was analyzed (F (3, 34)=19.95, P<0.05). Post hoc test demonstrated that rats subjected to incision reduced the number of entries in the center of the arena (OF), compared to sham rats (P<0.05), characterizing an anxiety-like effect, which was completely blocked by exposure to LAV EO (P<0.05; Figure 6C ).

Naltrexone caused a partial reduction in the antihyperalgesic effect of LAV EO.

**Figure 7:**
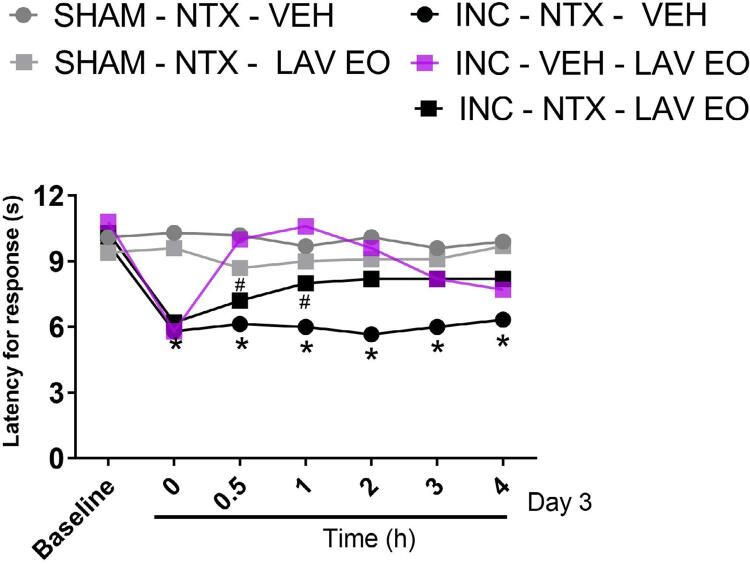
Naltrexone caused a partial reduction in the antinociceptive effect of LAV EO. ON day 3 after incision or sham surgery, rats received Naltrexone (NTX, 5 mg/kg, i.p.) or the corresponding vehicle (1 mL/kg, i.p.) and 15 minutes later they were exposed to LAV EO or VEH (both at 5%, for 30 minutes). The latency for responses to the heat stimulus was assessed before the surgery (Baseline), before the treatments (time 0) and at 30 minutes and 1 hour–intervals after the treatments The data are expressed as ± SEM (n=10), 2-way ANOVA with repeated measurements followed by the Bonferroni post hoc test. *P≤0,05 when compared to Sham-NTX-VEH, and #P≤0,05 when compared to INC-VEH-LAV OE

Two-way ANOVA with RM showed statistical difference in the treatment (F (5, 54)=28.01, P<0.05), time (F (6, 324)=69.15, P<0.05) and interaction among these factors (F (30, 324)=15.90, P<0.05). As can be seen in the Figure 7 , post hoc test showed that animals submitted to intraoral incision, differently of sham-operated group, when exposed to LAV OE by inhalation presented an increase in the latency time for response to heat stimulus in rats (P<0.05), indicative of antinociceptive effect. Systemic pretreatment with naltrexone (15 min before inhalation treatments) did not affect the response of the control groups (sham-operated and intraoral incision exposed to vehicle) (P>0.05), however it caused a partial reduction in the antihyperagesic effect of the LAV OE in animals subjected to incision (P<0.05).

## Discussion

The results of this study show, for the first time, that LAV EO by inhalation induced antinociceptive and anxiolytic-like effects in two different orofacial pain models. These findings support the clinical use of the LAV EO as an adjuvant in the control of pain and anxiety associated to orofacial procedures, encouraging the conduction of further studies that deepen the knowledge of other likely benefits that LAV EO inhalation can bring in other orofacial pain conditions.

There is increasing evidence that LAV EO and its major components, linalool and linalyl acetate, show antinociceptive and anti-inflammatory effects. *In vivo* experiments demonstrated that systemic administration of linalool reduced oedema induced by carrageenan in the rat hind paw,^31^ whereas *in vitro* it reduced nitric oxide release.^32^ Likewise, oral administration of LAV EO caused a reduction in carrageenan induced pleurisy and leucocyte infiltration and reduced rats’ ear oedema induced by croton oil. We highlight that the magnitude of the effect was similar after oral or topical application. Moreover, oral pre-treatment with LAV EO also reduced both phases of the response induced by formalin (2%) injected into the surface or rats’ hind paw. Herein, only the second phase of the paw formalin test was attenuated by LAV EO, but both phases of the orofacial formalin test were significantly reduced. The percentage of inhibition caused by inhalation of LAV EO in the phase I and II of the orofacial formalin test was 81% and 76%, respectively, and 47% for the phase II of the paw formalin test. The reason for this discrepancy remains to be investigated, but it may be related to the different administration routes. To our knowledge, there are no reports about the distribution of LAV EO after inhalation in rats or humans, but according to our data, inhalation may favor a better effect in the orofacial region. There is evidence that LAV EO may have a direct influence on rats’ trigeminal afferents, as well as may be rapidly transported directly to the brain from the nasal cavity along the olfactory and trigeminal nerves.^33^ These mechanisms could explain the rapid analgesic effects of LAV EO (i.e., immediately after the exposure) and the apparent greater susceptibility of the orofacial region. The nose to brain pathway has attracted attention in the clinical setting and in the market for many reasons, including rapid effect drugs delivered to the brain with minor side effects, and may be of special interest for the treatment of orofacial pain conditions. In addition to the administration route (oral x inhalation), another difference between the present study and the one by Da Silva and colleagues is the sex of animals used. There is increasing evidence about sex differences in the effect of analgesics drugs,^34^ and that would be a very interesting question to be explored in the effect of LAV EO.

Several reports of benefits of LAV EO inhalation in postoperative pain are seen in the clinical setting.^16 - 18^ In spite of that, it is difficult to draw a definitive conclusion, since the protocols, conditions, and endpoints vary enormously and many studies lack methodological quality. Thus, we highly recommend the development of high quality pre-clinical and clinical standardized studies that allow the inclusion of lavender aromatherapy in healthcare programs. Herein it was shown that previous exposure to LAV EO caused a rapid (i.e., within 30 minutes) and persistent (up to 3 hours) anti-hyperalgesic effect against facial heat and mechanical hiperalgesia in a model of post-operative pain. In line with these observations, in a model of inflammatory pain induced by Complete Freund Adjuvant (CFA) injection in the mice paw, it was shown that LAV EO inhalation for 30 minutes caused reduction of mechanical hyperalgesia.^7^ In this study, blockade of opioid receptors or cannabinoid CB2 receptors prevented the anti-hyperalgesic effect of the LAV EO, suggesting the involvement of both endogenous pain control systems in the analgesia induced by LAV EO. This finding is corroborated by previous reports showing that blockade of opioid receptors prevented the analgesic effect of linalool.^35 , 36^ In fact, on day 3 after incision surgery, pre-treatment of rats with naloxone 15 minutes before LAV EO exposure caused a partial reduction of its anti-hiperalgesic effect, reinforcing the idea that activation of opioid receptors contributes to LAV EO analgesia ( Figure 7 ). This observation could explain LAV EO effects in the first phase of the formalin response, which is susceptible to opioid analgesics.^25^ However, several additional mechanisms have been proposed to contribute to the therapeutic effect of LAV EO,^8 , 37^ which may also play a role in its analgesic effect.

The anxiolytic effect of LAV EO is well established and has been recently analyzed in two recent systematic reviews.^20 , 38^ The conclusion of both reviews is that LAV EO administered by oral route seems to provide benefit in anxiety management, but low quality and heterogeneity of the studies on this topic limited further interpretations. Moreover, there are several reports that LAV EO taken as silexan capsules promote anxiolytic effect in patients, which is comparable or even superior to gold standard treatments for anxiety.^39 , 40^ However, fewer studies assessed LAV EO anxiolytic effect after inhalation, and studies’ heterogeneity and risk of bias also limited the interpretation of findings. Herein it was shown that rats with persistent inflammatory pain showed anxiety-like behavior, which was significantly reduced by exposure to LAV EO by inhalation. The anxiolytic-like effect was assessed on the EPM and OF tests, which also revealed that LAV EO inhalation did not cause locomotor deficit, since the number of crossings in the OF and the number of entries in the closed arms of the EPM remained unchanged. On the other hand, LAV EO inhalation increased the number of entries in the center of the OF, and the number of entries and time spent in the open arms of the EPM of operated rats, indicating anxiolytic-like effect. These results corroborate previous evidence that LAV EO 5% by inhalation diminishes anxiety like-behavior in mice without affecting motor performance.^7 , 8^ In addition to the classical EPM parameters, ethological measures (i.e., risk assessment and head dip) were also assessed, allowing a deeper behavioral characterization.^29^ Risk assessment behavior is directed towards potential sources of danger and scanning of the environment for possible danger, while head dipping is an exploratory movement in open arms, so that the animal projects the head of the maze toward the floor. Both behaviors are considered sensitive indices of anxiety.^29 , 41^ We highlight that LAV EO attenuated these parameters, reinforcing the evidence of its anxiolytic-like effect after inhalation.

Evidence from pre-clinical studies indicates that mechanisms involved in the anxiolytic-like effect of LAV EO include inhibition of voltage dependent calcium channels, antagonism of NMDA receptors, inhibition of serotonin transporter, and activation of serotonin 5HT1A receptor.^8 , 37^ Likewise, all of these mechanisms may contribute to the analgesic effect of LAV EO, and may be of special importance in conditions in which pain and anxiety co-exist. The results of this study suggest that LAV EO may be useful in treating pain and associated anxiety, which agrees with some clinical reports.^16 , 42^ Moreover, studies conducted in humans do not clarify whether the anxiolytic effects of LAV EO are the result of action on the central nervous system (through the olfactory and limbic system) or whether the action is mediated peripherally.^10^ We point out that the olfactory nerve conducts information directly to the limbic system, a fundamental region in the processing of both pain and emotion. However, the precise mechanisms underlying LAV EO analgesic and anxiolytic effects, as well as the peripheral and central structures involved, remain to be elucidated.

## Conclusion

Our data suggest that LAV EO presents antinociceptive effects in orofacial pain models, corroborating clinical and non-clinical evidence. In addition, we demonstrated a consistent anxiolytic-like effect of LAV EO after inhalation in rats with persistent pain. These results corroborate few clinical observations that aromatherapy with LAV EO may provide benefit in pain and anxiety. We also suggest that the efficacy of LAV EO by inhalation should be further explored in non-clinical and clinical studies, since it represents a simple and inexpensive method that may be particularly useful in orofacial pain conditions.

### Key Findings

Lavender essential oil reduced both phases of the orofacial formalin test.

Lavender essential oil reduced heat hyperalgesia after oral mucosa incision.

Lavender essential oil reduced mechanical hyperalgesia after oral mucosa incision.

Lavender essential oil presented anxiolytic-like effect in rats after oral incision.
